# 影像表现不典型的肺硬化性肺细胞瘤的诊断与术中决策：1例报告并文献复习

**DOI:** 10.3779/j.issn.1009-3419.2026.101.22

**Published:** 2026-08-20

**Authors:** Xingyi XU, Hui XIAO, Xiong YE

**Affiliations:** 1 200080 上海，上海交通大学医学院附属第一人民医院呼吸与危重症医学科; 1 Department of Respiratory and Critical Care Medicine, Shanghai General Hospital, Shanghai Jiaotong University, Shanghai 200080, China; 2 201318 上海，上海健康医学院临床医学院; 2 School of Clinical Medicine, Shanghai University of Medicine & Health Sciences, Shanghai 201318, China

**Keywords:** 硬化性肺细胞瘤, 临床特征, 影像学, 术中冰冻诊断, 免疫组化, Sclerosing pneumocytoma, Benign lung tumors, Clinical features, Imaging, Intraoperative frozen section diagnosis, Immunohistochemistry

## Abstract

硬化性肺细胞瘤（pulmonary sclerosing pneumocytoma, PSP）是一种罕见的肺内良性肿瘤，多见于中年女性，术前确诊较困难，易误诊为恶性肿瘤。本文报道1例影像表现不典型的PSP患者，并对其研究现状进行文献回顾。患者女，68岁，因胸闷就诊，胸部计算机断层扫描（computed tomography, CT）示右肺中叶分叶状实性结节，大小约26 mm×22 mm，伴双肺门、纵隔及双侧腋下多发稍大淋巴结，术后病理证实为PSP。结合本例特点并复习相关文献，我们认为：当患者具有PSP的临床背景提示，且术中冰冻苏木精-伊红染色以单一实性或乳头状结构为主、表面细胞和间质细胞两类细胞难以辨认时，可考虑加做术中快速免疫组化，以提高诊断准确性，避免过度切除。

## Abstract (en)

Pulmonary sclerosing pneumocytoma (PSP) is a rare benign pulmonary tumor that predominantly occurs in middle-aged women. Its preoperative diagnosis remains challenging, and it is frequently misdiagnosed as a malignant tumor. Herein, we report a case of PSP with atypical imaging manifestations and review the current literature on this entity. A 68-year-old woman presented with chest tightness. Chest computed tomography (CT) revealed a lobulated solid nodule in the right middle lobe measuring approximately 26 mm × 22 mm, accompanied by multiple enlarged lymph nodes in the bilateral hilar, mediastinal, and axillary regions. The diagnosis of PSP was confirmed by postoperative histopathology. Based on the features of this case and review of the relevant literature, we suggest that when a patient presents with clinical features suggestive of PSP and intraoperative frozen-section hematoxylin and eosin staining predominantly shows a single solid or papillary architecture in which the surface and stromal cells are difficult to distinguish, intraoperative rapid immunohistochemistry may be performed to improve diagnostic accuracy and avoid excessive surgical resection.

硬化性肺细胞瘤（pulmonary sclerosing pneumocytoma, PSP）起源于不完全分化的II型肺泡细胞或者Clara细胞，是一种罕见的具有潜在恶性生物学行为的肺部良性肿瘤，好发于中年女性，生长缓慢。多数患者无特异性临床症状，常在体检时发现，影像学表现常与恶性肿瘤难以区分，确诊主要依赖术后病理学检查。手术是PSP的主要治疗方法，术后复发率低，预后良好。然而，PSP并非绝对良性，仍存在复发及远处转移的潜在风险。本文报告1例PSP并结合相关文献进行复习，以期为提高对该病的认识及临床诊疗水平提供参考。

## 1 病例资料

患者女，68岁，因“胸闷不适2天”入院。患者自2026年2月4日出现胸闷，无胸痛、呼吸困难、发热。胸部计算机断层扫描（computed tomography, CT）示右肺中叶实性结节影，大小约26 mm×22 mm，可见分叶（图1）。双肺门、纵隔及双侧腋下多发稍大淋巴结。本例术前未行^18^F-氟代脱氧葡萄糖正电子发射计算机断层显像/CT（^18^F-fluorodeoxyglucose positron emission tomography/CT, ^18^F-FDG PET/CT）检查，双肺门、纵隔及腋窝多发淋巴结亦未行病理学取材，其性质尚不能明确。基于CT检查结果，不能排除右肺肿瘤，入院后完善相关术前检查，于2026年2月9日全麻下行胸腔镜下肺叶部分切除术，术中冰冻切片快速病理检查结果提示为良性病变。术后苏木精-伊红（hematoxylin and eosin, HE）染色结果（图2）及免疫组化（图3）证实为PSP。免疫组化示：上皮膜抗原（epithelial membrane antigen, EMA）、细胞角蛋白（cytokeratin, CK）、CK7、甲状腺转录因子-1（thyroid transcription factor-1, TTF-1）、Napsin-A、孕激素受体（progesterone receptor, PR）、雌激素受体（estrogen receptor, ER）、核增殖抗原（Ki-67 proliferation index, Ki-67）2%+、细胞角蛋白CAM5.2阳性。术后4个月，电话随访患者，未诉明显不适。该病例报道已获患者知情同意。患者相关临床资料、影像学资料及病理图片均已进行匿名化处理。

**图1:**
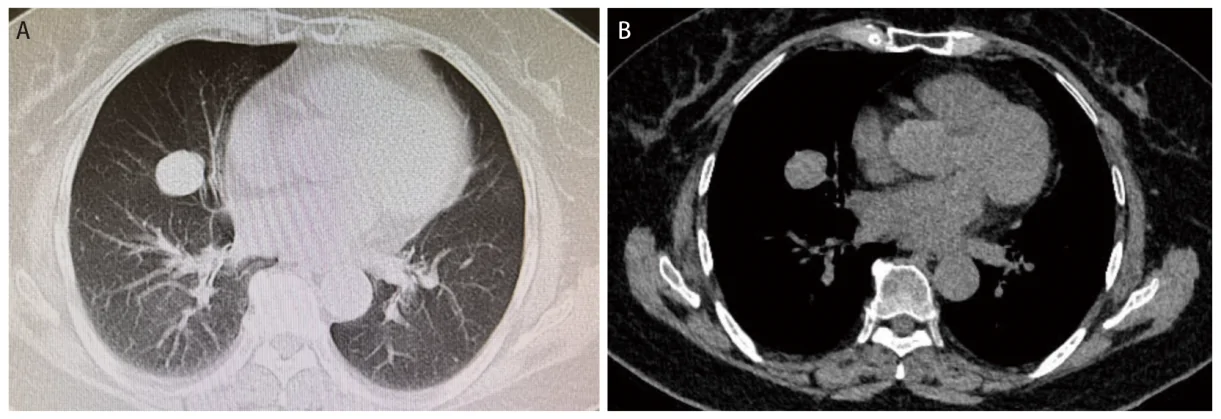
患者胸部CT提示右肺中叶实性结节影，大小约26 mm×22 mm，可见分叶征。A：手术前胸部CT检查（肺窗）；B：手术前胸部CT检查（纵隔窗）。

**图2:**
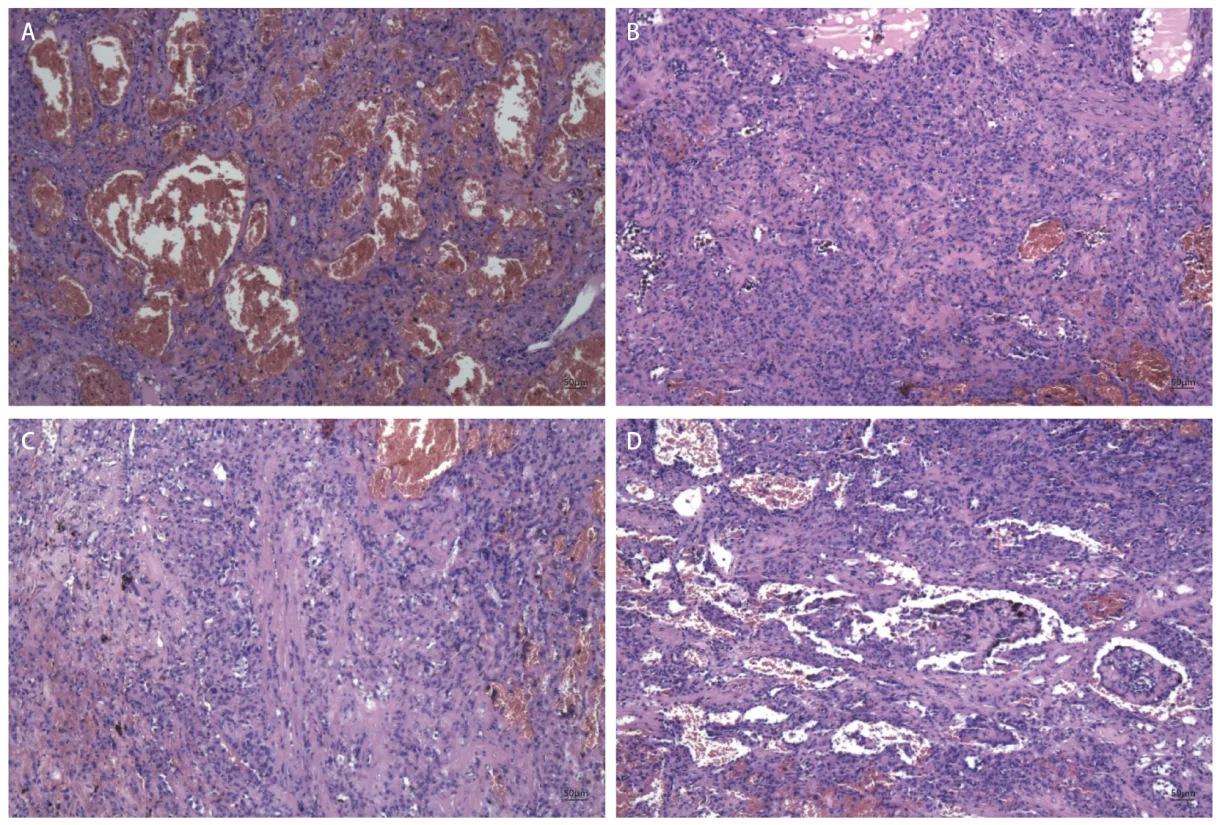
HE染色结果。本例患者镜下可见4种典型的组织结构：出血性结构（A）、实性结构（B）、硬化性结构（C）与乳头状结构（D），以出血性结构为主。

**图3:**
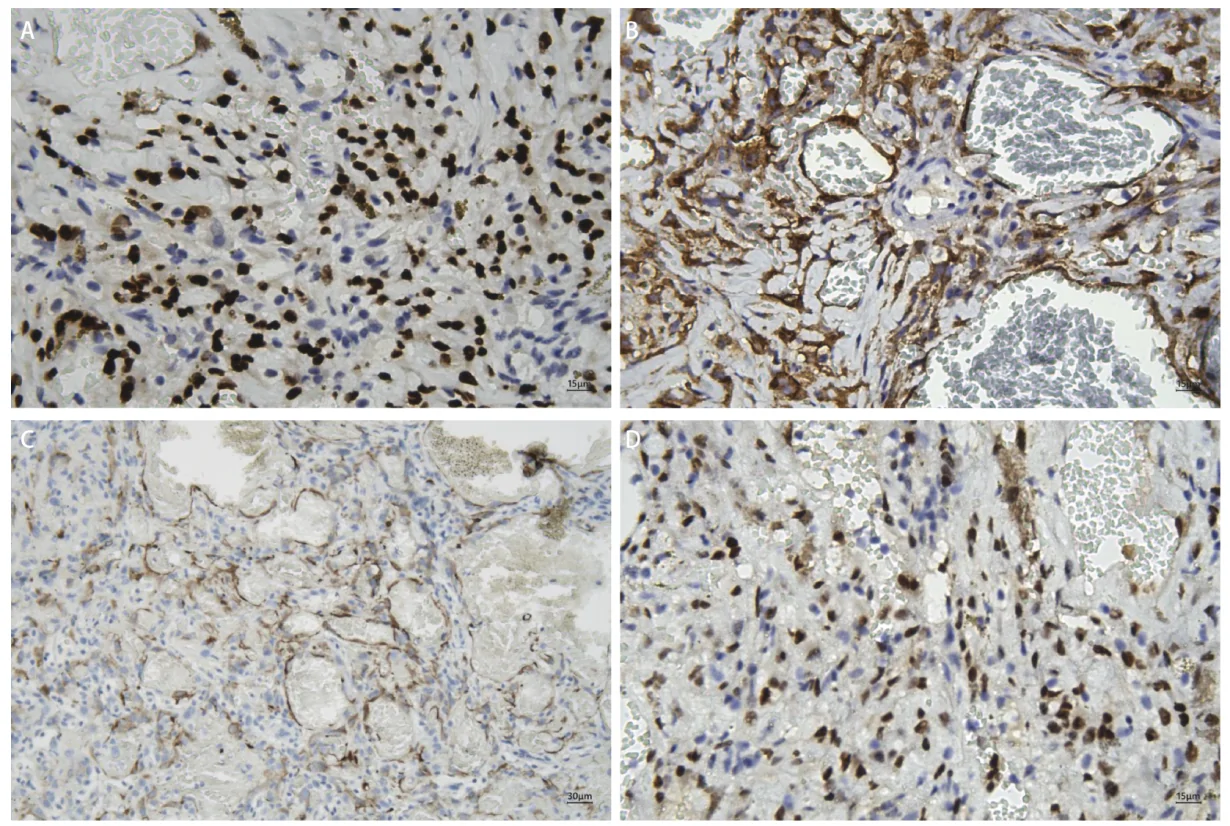
免疫组化检测结果。A：表面细胞及间质细胞TTF-1阳性；B：表面细胞及间质细胞EMA阳性；C：表面细胞CK阳性；D：间质细胞PR阳性。

## 2 讨论

PSP是一种临床罕见的具有恶性潜能的肺部良性肿瘤，1956年由Liebow首次报道并命名为“硬化性血管瘤”，2015年世界卫生组织（World Health Organization, WHO）肺肿瘤分类正式更名为现名^[1]^。PSP多见于中年女性，多数患者无明显症状，常在体检或因其他原因行影像学检查时发现，少数患者可出现咳嗽、咳痰、胸痛或咯血等非特异性呼吸道症状。本例患者因胸闷就诊，以胸闷为首发表现者文献报道较少。

PSP的典型CT表现为肺内单发、圆形或类圆形、边界清晰的软组织密度影，平均直径2.0-2.5 cm。特征性CT征象包括：边缘假包膜征、血管贴边征、空气新月征、晕征^[2]^。本例患者术前CT右肺中叶可见实性结节（图1），呈分叶状，未见明确的典型征象。因此，影像学表现与肺癌存在重叠，术前难以作出明确诊断。提示临床在遇到中年女性肺部孤立性结节时，即使病灶表现为分叶、边缘毛糙等可疑恶性征象，仍应将PSP纳入鉴别诊断。

^18^F-FDG PET/CT为PSP与肺癌的鉴别提供补充信息。研究^[3-5]^报道PSP的FDG摄取量与瘤体直径呈线性正相关（*r*=0.642-0.754），直径>2 cm时最大标准化摄取值（standardized uptake value maximum, SUVmax）可显著升高，易被误判为恶性。值得警惕的是，部分PSP及转移性淋巴结可呈现高FDG摄取，摄取模式类似恶性肿瘤^[5]^。文献^[6]^提示对于不典型影像表现者，延迟期SUVmax可能提供更多鉴别信息。

PSP的确诊主要依赖组织病理学及免疫组化检查，其典型特征可概括为“两种细胞、四种结构”^[7]^。表面细胞形态类似II型肺泡上皮细胞，间质细胞呈椭圆形或多边形、胞质淡染；4种组织结构包括乳头状结构、实性结构、出血性结构和硬化性结构。多数病例由多种结构混合构成，约52.7%的病例呈2-3种结构，17.1%同时具有4种结构，另有30.3%以单一结构为主^[8]^。本例患者镜下可见4种结构，且以出血结构为主（图2）。

PSP组织结构和细胞成分的异质性是术中冰冻诊断困难的重要原因。当乳头状结构占优势时，易与肺腺癌混淆^[9]^，实性结构突出时则需与类癌等肿瘤鉴别^[10]^。加之冰冻切片取材范围有限且冰晶形成可影响细胞形态，使得表面细胞与间质圆形细胞在HE染色中有时难以准确区分。此外，约1/3的病例可出现多核瘤巨细胞、黏液变性、硬化性乳头及间质细胞梭形变等变异，需与转移性肾透明细胞癌、软组织梭形细胞肿瘤等鉴别^[11]^。故对不典型病例推荐多点取材并结合免疫组化。文献^[12]^曾报道1例初诊时因未行TTF-1检测，仅凭荧光原位杂交临界信号误诊为神经营养因子受体酪氨酸激酶（neurotrophin receptor kinase, *NTRK*）重排梭形细胞肿瘤，直至3年后复发再手术，方经免疫组化及分子检测修正诊断为PSP。因此，对肺内梭形细胞肿瘤应将PSP纳入鉴别诊断，并重视TTF-1检测。

免疫组化检查有助于识别两类肿瘤细胞并明确诊断^[13]^。两类细胞通常均表达TTF-1和EMA；表面细胞表达CK和Napsin-A，间质细胞主要表达Vimentin；Ki-67增殖指数多低于5%。部分病例表达ER或PR，此特征或与其女性高发倾向有关，且与肺腺癌（ER低表达）形成鉴别点。本例患者两类细胞TTF-1、EMA均阳性，表面细胞CK、Napsin-A阳性，间质细胞PR、ER阳性，符合典型PSP免疫表型。

针对术中冰冻切片以单一结构或单一细胞成分为主、两种细胞难以辨认的病例，可在形态学评估的基础上，酌情进行术中快速免疫组化检测。TTF-1、pan-CK和Vimentin联合标记可从细胞来源和分布模式方面提供辅助依据：两种细胞均可表达TTF-1，CK主要标记表面细胞，Vimentin主要标记间质细胞。对于检测结果仍不能支持明确诊断的病例，宜作描述性诊断，待常规石蜡切片及完整免疫表型进一步确认，以降低误诊和扩大切除的风险。

分子研究显示，AKT丝氨酸/苏氨酸激酶1（AKT serine/threonine kinase 1, *AKT1*）p.E17K突变是PSP较常见的分子事件，通过激活磷脂酰肌醇3-激酶/AKT（phosphatidylinositol 3-kinase/AKT, PI3K/AKT）信号通路可驱动肿瘤发生发展^[14]^。梭形细胞为主型病例的复发灶中同样检出*AKT1 *p.E17K突变^[12]^，进一步印证了该突变在PSP不同形态亚型中的普遍性。典型的PSP呈惰性经过，单一*AKT1*突变通常不足以驱动恶性进展，部分不典型或具有转移行为的病例还可出现肿瘤蛋白53（tumor protein 53, *TP53*）、磷脂酰肌醇-4,5-二磷酸3-激酶催化亚基α（phosphatidylinositol-4,5-bisphosphate 3-kinase catalytic subunit alpha, *PIK3CA*）、富含AT碱基互作结构域蛋白1A（AT-rich interaction domain 1A, *ARID1A*）、β-连环蛋白1（catenin beta-1, *CTNNB1*）等基因异常^[15]^。然而，相关分子改变目前主要用于机制研究，PSP的常规诊断仍以组织形态和免疫表型为基础。

手术是PSP的核心治疗手段。对于能够完整切除的局限性病灶，楔形切除或肺段切除相较于肺叶切除通常可以缩短术后引流管留置时间及住院时间，并有助于保留肺功能^[16]^。淋巴结处理方面，现有文献不支持将其等同于恶性肿瘤进行过度干预，建议仅切除术前影像或术中触及的肿大淋巴结^[17]^。本例患者术中快速病理报告为良性，遂仅行右肺中叶部分切除，未行淋巴结清扫。

PSP总体预后良好，仅0.8%-4%发生淋巴结转移。已报道的危险因素包括男性、年龄≤41岁及肿瘤直径≥36 mm^[18,19]^。转移机制可能与上皮-间质转化（epithelial-mesenchymal transition, EMT）相关，转移灶主要由圆形细胞构成^[20]^。值得注意的是，即使发生淋巴结转移，经长期随访，多数患者仍无复发或远处播散，预后未受显著影响。

综上所述，PSP是一种总体预后良好但组织学及影像学表现具有异质性的肺部肿瘤。对于中年女性的孤立性肺结节，即使表现为分叶、边缘毛糙等疑似恶性征象，仍应将PSP纳入鉴别诊断；术前选择影像随访、穿刺活检或手术，应综合病灶大小及动态变化、恶性影像特征、患者危险因素和组织学取材条件进行判断。病理诊断应以“两种细胞、四种结构”为基础，并结合免疫组化结果综合判断；对于术中冰冻切片结构单一、两种细胞难以辨认的病例，可联合应用TTF-1、CK和Vimentin辅助诊断，仍不能明确时应避免仓促扩大切除范围。治疗应以完整切除和最大程度保留肺功能为原则，并对男性、年轻及肿瘤较大的患者适当加强随访。
